# Mathematical-model-guided development of full-thickness epidermal equivalent

**DOI:** 10.1038/s41598-018-36647-y

**Published:** 2018-12-20

**Authors:** Junichi Kumamoto, Shinobu Nakanishi, Mio Makita, Masaaki Uesaka, Yusuke Yasugahira, Yasuaki Kobayashi, Masaharu Nagayama, Sumiko Denda, Mitsuhiro Denda

**Affiliations:** 1 Research Institute for Electronic Science, Hokkaido University, Sapporo, Japan; 2 Shiseido Global Innovation Center, Yokohama, Japan; 3 Graduate School of Science, Hokkaido University, Sapporo, Japan; 4 Center for Simulation Sciences, Ochanomizu University, Tokyo, Japan

## Abstract

Epidermal equivalents prepared with passaged keratinocytes are typically 10–20 μm thick, whereas intact human epidermis is up to 100 μm thick. Our established mathematical model of epidermal homeostasis predicted that the undulatory pattern of the papillary layer beneath the epidermis is a key determinant of epidermal thickness. Here, we tested this prediction by seeding human keratinocytes on polyester textiles with various fiber-structural patterns in culture dishes exposed to air, aiming to develop a more physiologically realistic epidermal model using passaged keratinocytes. Textile substrate with fiber thickness and inter-fiber distance matching the computer predictions afforded a three-dimensional epidermal-equivalent model with thick stratum corneum and intercellular lamellar lipid structure. The basal layer structure was similar to that of human papillary layer. Cells located around the textile fibers were proliferating, as indicated by BrdU and YAP (Yes-associated protein) staining and expression of melanoma-associated chondroitin sulfate proteoglycan. Filaggrin, loricrin, claudin 1 and ZO-1 were all appropriately expressed. Silencing of transcriptional coactivator YAP with siRNA disturbed construction of the three-dimensional structure. Measurement of trans-epidermal water loss (TEWL) indicated that the model has excellent barrier function. Our results support the idea that mathematical modeling of complex biological processes can have predictive ability and practical value.

## Introduction

Experimental models of human epidermis are useful research tools for basic studies of skin biology and functional mechanisms, as well as for the development of transdermally administrable medicines and safety testing of cosmetics and other products^1^. However, epidermis has a complex structure consisting of multiple layers^2^ and is not adequately mimicked by many current models^1^. For example, epidermal models prepared with passaged keratinocytes are usually non-physiologically thin (10–20 μm), although thick epidermal equivalents (around 100 μm) have been constructed with primary keratinocytes^3^. However, it is difficult to obtain primary keratinocytes with consistent properties in large amounts.

We have established methodology for simulating epidermal homeostasis^4–6^, employing a mathematical model composed of keratinocytes generated from stem cells distributed in the basal layer, and taking into account dynamic cellular processes in the epidermis, such as migration and differentiation, as well as the interaction of intracellular Ca^2+^ dynamics with differentiation. Our previous numerical simulations successfully reproduced a spatially and temporally stable epidermal structure with sufficient thickness and a flat stratum corneum/suprabasal layer interface^6^. Calculations suggested that the epidermal structure and thickness are greatly influenced by the spatial distribution of stem cells and the structure of the basement membrane on which they are seeded. When we applied sinusoidal modulation of the basement membrane shape and systematically changed the amplitude and wavelength, we found that formation of a thick and stable epidermal structure required basement membrane undulations with large amplitude and short wavelength^6^. This result indicated that the undulatory pattern of the papillary layer, which lies at the top of the dermis immediately below the epidermis, is critical for constructing an epidermal model with physiological thickness. However, one of the most important roles of the epidermis is its water-impermeable barrier function, which requires a sufficiently thick stratum corneum. Since our previous model^6^ did not take into account flattening of corneocytes, it could not properly simulate the thickness of stratum corneum. Here, we first established a new three-dimensional numerical simulation model that incorporates flattening of corneocytes to form a stratum corneum sheet. Then, based on the computational predictions of the improved simulation model, we examined whether a full-thickness three-dimensional epidermal-equivalent model that included stratum corneum and intercellular lamellar lipid structure, which are essential for skin barrier function, could be obtained by seeding passaged human keratinocytes on an undulating surface consisting of polyester textile with an appropriate fiber pattern in culture dishes exposed to air. Our results represent a proof-of-principle of the value of this methodology for establishing high-quality epidermal-equivalent models with passaged keratinocytes, and also indicate the importance of substrate structure for epidermal development. They also support the idea that mathematical modeling of complex biological processes can have predictive ability and practical value.

## Results

### Structure of epidermal-equivalent models grown on patterned textile substrates

**Figure 1:**
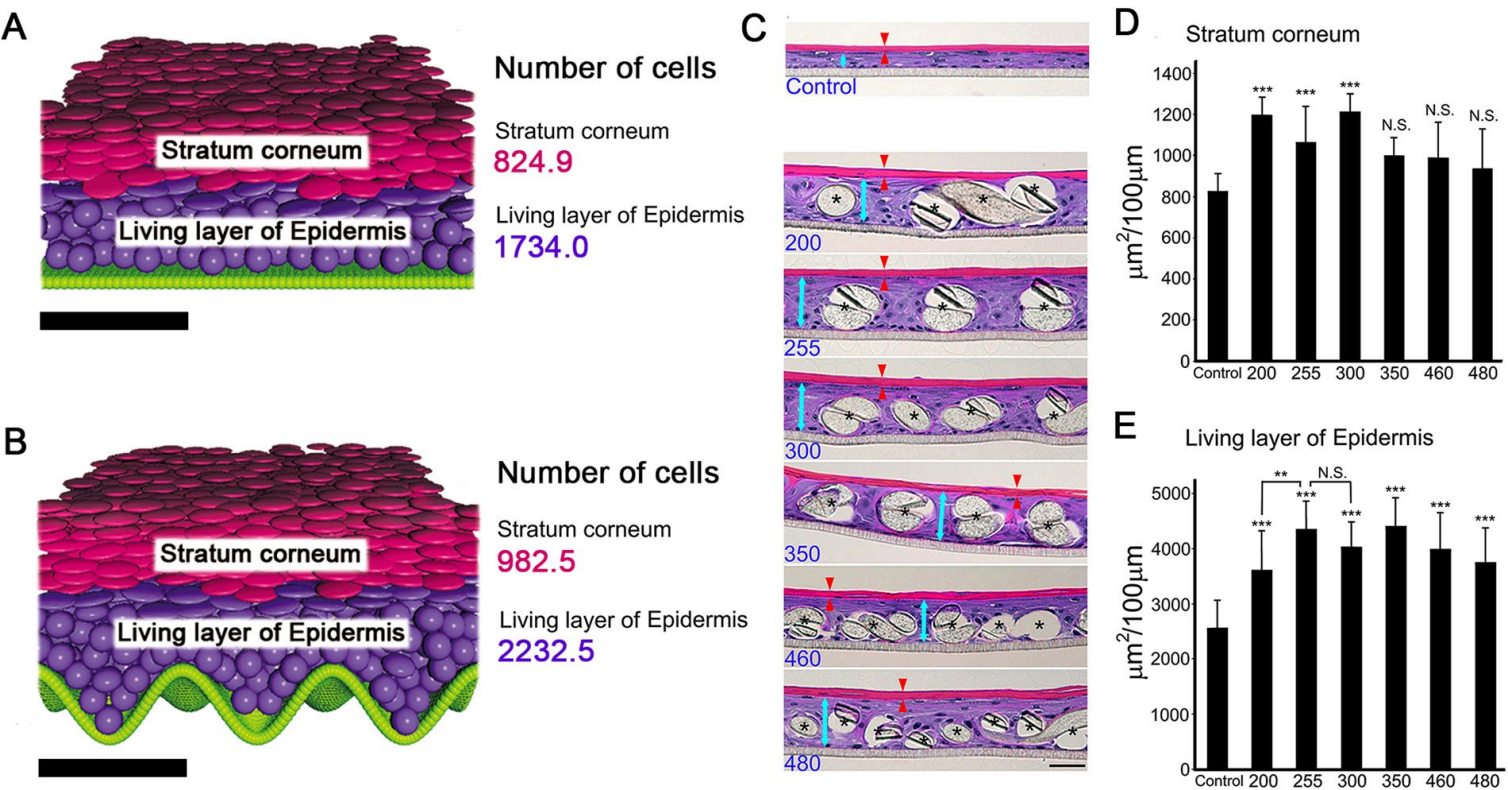
Results of computer simulations of epidermal growth on a flat basement membrane (**A**) and a sinusoidal basement membrane. (**B**) ROI: x: 300 μm, y: 300 μm. Numbers of cells in stratum corneum and living layer of each models are indicated on the right. Basement membrane, green; living keratinocytes, purple; cornified cells, red. Bars = 100 μm. Representative images of epidermal-equivalent models. (**C**) Number at the bottom of each image is the substrate textile number (see Table 1). Living layers are indicated by blue arrows, and stratum corneum by red arrows. Asterisks indicate cross sections of fibers. Bars = 50 μm. (**D**) Quantified results of stratum corneum area per 100 μm of section (n = 4–6, ANOVA F value = 10.489123, P < 0.0001). (**E**) Quantified results of living layers area per 100 μm of section (n = 4–6, ANOVA F value = 19.154106, P < 0.0001). ***P < 0.0001, Mean + SD.

The results of the new three-dimensional computer simulation and the cultured epidermal models are illustrated in Fig. 1A–E. Compared with the result obtained using a flat basement membrane (Fig. 1A), the stratum corneum and living layer were thicker on the sinusoidal basement membrane (Fig. 1B). The numbers of cells in the stratum corneum and the living layer of the epidermis in the two cases are shown on the right.

To validate the simulation results, we attempted to construct a thick epidermal model with passaged keratinocytes by using an appropriately patterned substrate. For this purpose, we seeded keratinocytes on a series of textile samples with different textures in culture dishes. For comparison with the textile pattern parameters (Table 1, Supplement Fig. 1), we also measured the corresponding values of human papillary layer, which exhibited a rete ridge height (corresponding to fiber thickness) of 51 μm and an undulation interval (fiber interval) of 105 μm (n = 13, abdominal skin, average age of subjects 36.3 years).

**Table 1:** Properties of polyester textile samples used as substrates.

Identification	Fiber	Inter-fiber
No. of textile	thickness (μm)	interval (μm)
74	205	354
86	165	301
125	122	201
160	100	164
200	73	127
255	60	101
300	51	87.6
350	60	75.2
460	41	57.5
480	55	52.9

Representative microscopic hematoxylin and eosin (H&E)-stained images of the grown epidermal models are shown in Fig. 1C. The results of quantification of the living layer of epidermis and stratum corneum are shown in Fig. 1D,E, respectively (no epidermal sheet developed on samples #74, #86, #125, and #160). Textiles #200, #255 and #300 afforded significantly thicker stratum corneum than the control (no textile). The living layer of epidermis on textile #255 was thicker than that on textile #200 (statistically significant) or #300 (not significant). Therefore, we focused on textile #255 for further study.

### Integrity of the epidermal-equivalent model and location of proliferating cells

**Figure 2:**
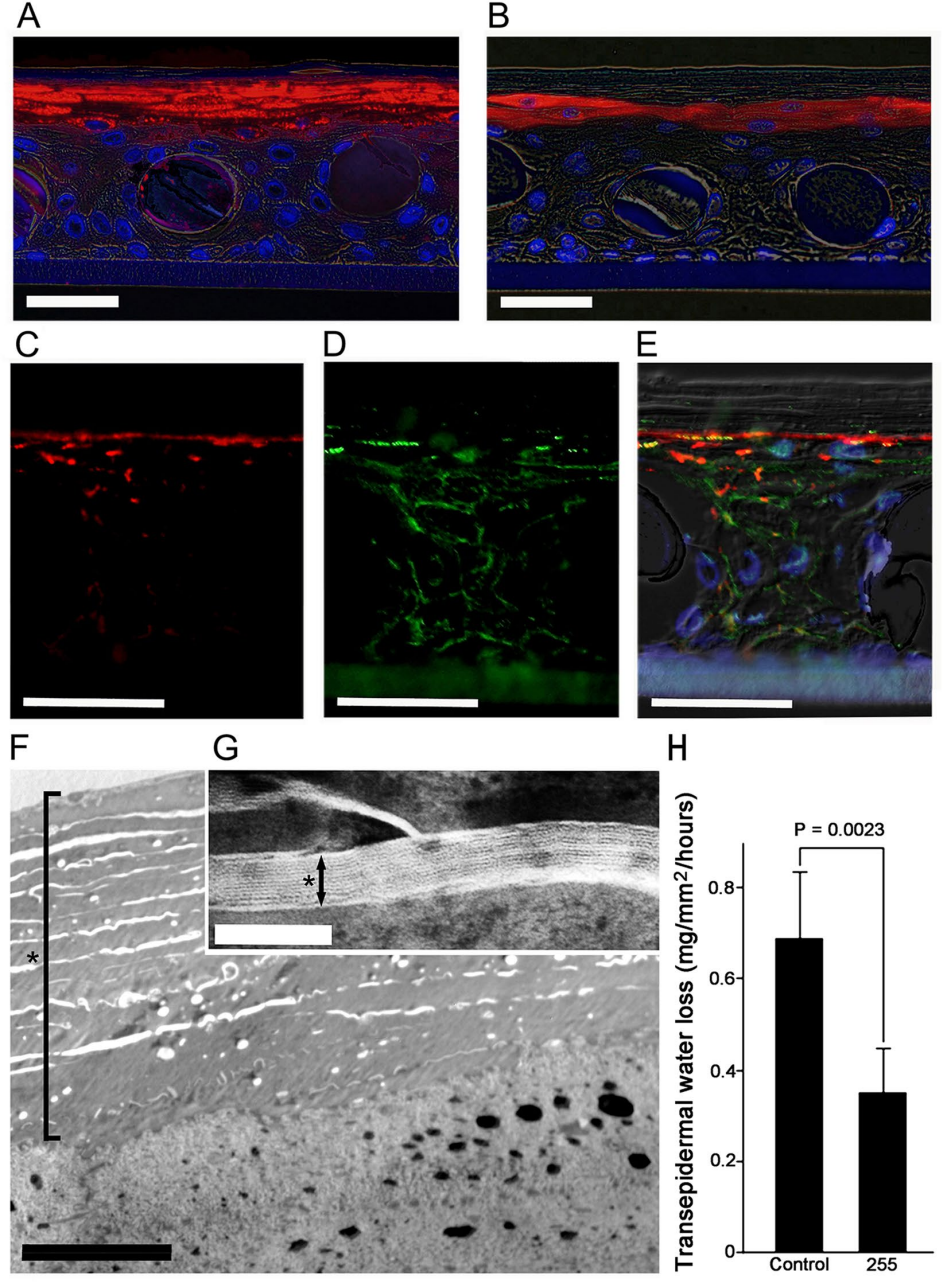
Immunostaining and electro-microscopic observation. Differentiation markers filaggrin (**A**) and loricrin (**B**) are expressed at the uppermost layer of epidermis. Tight junction markers ZO-1 (**C**) are expressed at the upper layer of the epidermis and claudin 1 (**D**) are expressed at the cell membrane throughout the epidermis. (**E**) Merged image of **C** and **D**. Bars = 20 μm. (**F**,**G**) Electron-microscopic images of model grown on textile #255. Thick stratum corneum was observed (F, black asterisk; bar = 5 μm) and contained intercellular lipid bilayer structure (**G**, black asterisk, bar = 100 nm). Transepidermal water loss of control and epidermal models grown on textile #255 (H). Models grown on textile #255 showed significantly lower TEWL. Mean + SD. n = 6.

Structural proteins filaggrin, loricrin, claudin 1 and ZO-1 play key roles in epidermis^7,8^ so to confirm that they were appropriately expressed, we carried out immuno-histochemical studies of epidermal models grown on textile #255. As shown in Fig. 2, differentiation markers filaggrin (Fig. 2A) and loricrin (Fig. 2B) and tight-junction markers ZO-1 (Fig. 2C) were all appropriately expressed at the upper layer of the epidermis. Claudin 1 was expressed in the cell membrane throughout the epidermis (Fig. 2D). In addition, electron-microscopic images revealed thick stratum corneum (Fig. 2F, black asterisk) containing intercellular lipid bilayer structure (Fig. 2G, black asterisk), suggesting that the model would show effective barrier function. Immunohistochemical studies of the model grown on textile #300 and the control are shown in supplement Fig. 2. ZO-1 and claudin 1 were expressed in both cases, though expression of ZO-1 seemed weaker in the model grown on textile #300 than in that grown on textile #255.

To confirm this, we measured trans-epidermal water loss (TEWL) of control and epidermal models (Fig. 2H). Compared with the control, epidermal models grown on textile #255 showed significantly lower TEWL, indicating that they have greater barrier function. In the present proof-of-concept study, we focused on TEWL to evaluate the skin equivalent model because it is widely used as a parameter of epidermal water-impermeable barrier function, which is critical for the present purpose.

**Figure 3:**
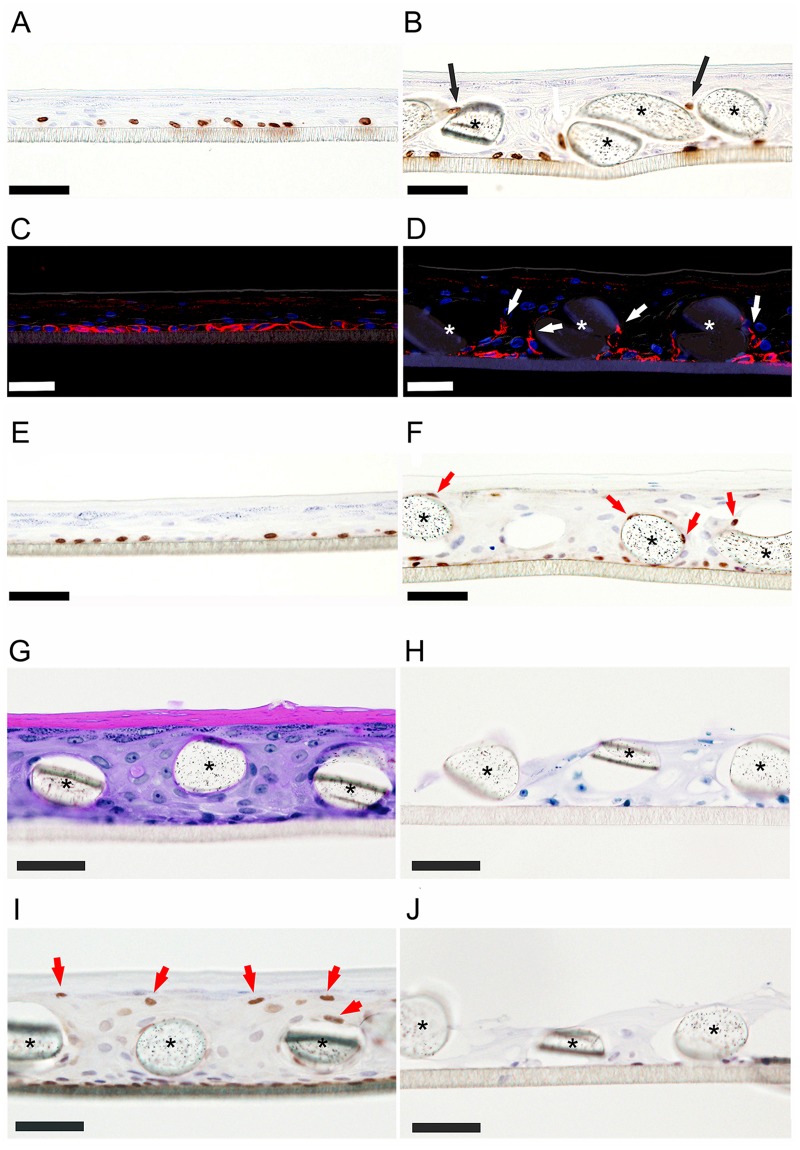
Proliferation markers and role of YAP. (**A**,**B**) Anti-BrdU staining (**A**, control; **B**, #255 textile, black asterisks indicate cross sections of fibers.). In the control, proliferating cells appear only at the bottom. Proliferating cells appear at fiber surfaces in **B** (black arrows). (**C**,**D**) Anti-MCSP immunostaining (**C**, control; **D**, #255 textile, white asterisks indicate cross sections of fibers). MCSP-positive cells appear on fibers (white arrows). (**E**,**F**) Anti-YAP immunostaining (**E**, control; **F**, #255 textile). YAP-positive cells appear on fibers (red arrows). (**G–J**) Effects of YAP siRNA (#255 textile). (**G**,**H**) H&E staining. (**G**) Control shows thick epidermis and stratum corneum. (**H**) YAP siRNA application disturbed structure formation. (**I**,**J**) Anti-YAP immunostaining. (**I**) YAP is expressed on fibers in control (red arrows). (**J**) Very little YAP expression was observed in YAP siRNA-applied sample. Black asterisks indicate cross sections of fibers. Bars = 50 μm.

Anti-BrdU staining revealed that proliferating cells were present only at the bottom in the control (Fig. 3A no textile), whereas proliferating cells were also observed on fibers of the textile substrate in the experimental model (Fig. 3B, black arrows). We also examined expression of melanoma-associated chondroitin sulfate proteoglycan (MCSP), which plays a role in stabilizing cell-substratum interactions^9^. Anti-MCSP immunostaining revealed positive cells on fibers (Fig. 3D, white arrows), supporting the idea that at least some cells on fibers had proliferative ability. Supplement Fig. 3 shows the results of double immunohistochemical staining with anti-BrdU and K14, a basal-layer marker, of the model grown on textile #255 and the no-textile control. Co-expression of BrdU and K14 was observed on the top of the fibers in the model, suggesting that proliferating cells may recognize the top of the fibers as a basal layer.

### Role of Yes-associated protein (YAP) in thick structure development

We next examined the role of the key transcriptional regulator YAP in the present model by employing siRNA. In control epidermis, YAP was localized only at the basal layer (Fig. 3E), while in the textile-grown model, YAP was expressed around fibers (Fig. 3F, red arrows). Representative images are shown in Fig. 3G–J. Application of YAP siRNA markedly disturbed the formation of three-dimensional structure, as can be seen by comparison of H&E staining of control and YAP siRNA-applied textile-grown samples (Fig. 3G,H respectively). In control siRNA-treated epidermis, YAP was expressed around fibers (Fig. 3I), while little expression was observed in YAP siRNA-treated samples (Fig. 3J). Application of YAP siRNA did not influence proliferation of keratinocytes in monolayer culture (data not shown). YAP siRNA application also disturbed construction of three-dimensional structure in the control model (Supplement Fig. 4). The precise role of YAP in construction of the thicker epidermal model remains to be established.

## Discussion

The *in vitro* results confirmed our in-silico prediction that undulation of the basement membrane is critical for formation of thick stratum and epidermal living layer. Indeed, the living layer of epidermis and the stratum corneum in the epidermal model were thickest when we used textile substrate having an undulation pattern whose spatial scale was comparable to that predicted to be most effective by the computer simulation. Notably, this pattern is also similar to that of the human skin papillary layer in 36-year-old healthy subjects. We also observed BrdU staining and expression of MCSP on cells located on textile fibers in our model, suggesting that cell contact with the textile fibers might promote proliferation. The size and geometry of cells contribute to construction of tissue structure, because they influence intracellular biochemical patterns in multi-cellular systems^10^. In addition, the spatial environment of bronchial epithelial cells influences proliferation rate^11^. Our results are thus consistent with the idea that the spatial features of the basal layer of epidermis play a key role in epidermal structure development and homeostasis, and therefore that the substrate textile pattern is crucial for construction of a thick epidermal model in our system. Further, since rete ridge height decreases with aging^12^ and epidermal permeability barrier recovery becomes slower with aging^13^, it will be interesting to investigate whether aging-related changes of the undulation pattern of the basal layer are associated with impaired epidermal structure.

Yes-associated protein (YAP) is a transcriptional regulator associated with proliferation^14^. It is important in constructing three-dimensional body shape^15^ and in mechanotransduction^16^. In epidermis, YAP is involved in stem cell proliferation^17^, terminal differentiation, epidermal permeability-barrier formation^18^ and wound healing^19^. Our findings indicate that YAP may also play a critical role in the development of thick three-dimensional structure in our epidermal-equivalent model, though the mechanism involved remains to be determined.

This work has confirmed the practical value of our mathematical model for guiding the establishment of an epidermal equivalent model with a thick epidermal living layer and thick stratum corneum using passaged keratinocytes. Although other methods are available to construct 3D epidermal equivalent models, as recently reviewed^20^, they have limitations. Various scaffold-based 3D models are available, but all of them require a hydrogel scaffold, which our model does not. A scaffold-free 3D methodology has been described, but can only construct micro-scale models, so its usefulness is limited.

Although we focused on TEWL to evaluate our model, and did not carry out further evaluation of the barrier function, we believe our work provides a proof-of-concept of new methodology for the production of physiologically realistic epidermal-equivalent models having a full-thickness epidermal layer that displays signs of differentiation, tight junctions and stratum corneum with intercellular lipid bilayer structure, using passaged keratinocytes. Here, we used keratinocytes after three passages, which provided a 512-fold increase of cell number. This should make it possible to construct low-cost epidermal-equivalent models while retaining high quality, although further optimization and validation studies will be necessary. These findings nicely illustrate the utility of in-silico modeling of complex biological processes, since model parameters and conditions can be easily and inexpensively modified in computer simulations.

## Materials and Methods

The mathematical model and computer simulation methodology are described in the supplement “Computer simulation”^21^.

### Keratinocyte culture

The basic methodology for constructing the skin equivalent model was as described previously^3^. Normal human epidermal keratinocytes collected from neonate foreskin (NHEKs) from Kurabo (Osaka, Japan) were cultured in Epilife-KG2 (Kurabo) containing 0.06 mM Ca^2+^ in 10 cm dishes and passaged three times. Then, 2.2 × 10^5^ keratinocytes/500 μl CnT Prime medium (CELLnTEC, Berne, Switzerland) were plated on 12-well Millicells with 0.4 µm pore size PET hanging inserts (bore diameter 12 mm, Millipore, Billerica, MA). The inserts were precoated with CellStart (Invitrogen Life Technologies, Carlsbad, CA) in a 50X dilution of DPBS. CnT Prime (CELLnTEC, Bern, Switzerland) (1 ml) was added to each well. At 72 hours after seeding (day 3) the medium was switched to CnT-PR-3D differentiation medium (CELLnTEC, Bern, Switzerland) both inside and outside the inserts. Cultures were submerged in differentiation media for 16 hours and then lifted to the air-medium interface by removing excess medium from the insert and reducing the volume of differentiation media on the outside to 500 µl. Cultures were fed daily with 500 µl of differentiation media for 9 days and then harvested. To evaluate proliferative cells, BrdU was applied in the medium 24 hours before the harvest.

### Polyester textile substrates

Polyester textile (12-mm diameter; Clever, Toyohashi, Japan) with a variety of fiber patterns was fixed with Rocktite 3554 (Henkel, Düsseldorf, Germany) and RTV118 (Momentive, New York, USA) to the bottom of the inserts, then pre-coated with CellStart, and seeded with keratinocytes. Incubation was conducted as above. The characteristics of the textiles used are summarized in Table 1.

### Evaluation of fiber patterns in textiles and normal human skin

Fiber patterns of textiles and normal human skin were evaluated as illustrated in Supplement Fig. 1. Human tissues (non-sunexposed area from abdomen, n = 13; average age of subjects 36.3 years), obtained with informed consent following plastic surgery, were purchased from Biopredic International (Rennes, France) via KAC Co., Ltd. (Kyoto, Japan). This study was approved by the ethics committee of Shiseido, and was conducted in accordance with the guideline of the National Institute of Health.

### YAP siRNA application

One day before seeding cells onto 12-well Millicells, the cells were grown to 80% confluency (approximately 2~3 × 10^6^ cells), and transfected with 20 nM scramble control or YAP siRNA (GE Dharmacon, Lafayette, CO, USA) using the transfection reagent RNA iMAX (Thermo Fisher Scientific, Waltham, MA USA) in OptiMem (Thermo Fisher Scientific) as described in the manual. Scramble control: ugguuuacaugucgacuaa, ugguuuacauguuguguga, ugguuuacauguuuucuga and ugguuuacauguuuuccua. YAP: gcaccuaucacucucgaga, ugagaacaaugacgaccaa, ggucagagauacuucuuaa, ccaccaagcuagauaaaga (GE Dharmacon, Lafayette, USA).

Total RNA from human keratinocytes was isolated using and RNeasy mini kit (QIAGEN, Hilden, Germany) for quantitative real-time PCR (RT-PCR). Complementary DNA (cDNA) synthesis from 1 μg of total RNA was performed using SuperScript VILO Master Mix (Invitrogen, Carlsbad, USA). The PCR reactions were performed using LightCycler 480 Probes Master (Roche, Basal, Switzerland), cDNA and specific primer pairs: GAPDH: forward, gaaggtgaaggtcggagtc and reverse, gaagattggtgatgggatttc; YAP: forward, cccagatgaacgtcacagc and reverse, ttcccatccatcaggaagag, on an LightCycler 480 System II (Roche, Basel, Switzerland). Results were normalized with the GAPDH gene and showed that YAP siRNA decreased YAP expression by 87 ± 2%.

### Histology

For hematoxylin and eosin (H&E) staining, anti-bromodeoxyuridine (BrdU) antibody or anti-YAP antibody staining, samples were fixed with 4% paraformaldehyde in PBS, embedded in paraffin, and sectioned at 3 μm. For the evaluation of areas of stratum corneum and living layer of the epidermis, we constructed 4–6 samples sections per textile. We acquired 3 images per one section and took the average value, and we used 3–5 sections from each condition. Image-J software was used to remove fiber cross-section areas from the images of the H&E-stained sections, and to evaluate the remaining area of the living layer of the epidermis, and area of stratum corneum.

For immunostaining of filaggrin, loricrin, and MCSP, samples were fixed in acetone at −20 °C for 30 min, embedded in paraffin and sectioned at 3 μm for staining. For tight junction markers, 6 μm frozen sections were fixed in methanol at −20 °C for 30 min.

Primary antibodies were rabbit polyclonal anti-filaggrin (1/300, # sc-30229, Santa Cruz, Dallas, USA), rabbit polyclonal loricrin (1/500, # PRB-145P, Biolegend, San Diego, USA), mouse monoclonal-anti-NG2/MCSP (1/400, # MAB2585, R&D Systems, Minneapolis, USA), rabbit polyclonal anti-claudin-1 antibody (1/500, #51–9000, Invitrogen, Carlsbad, USA) and mouse monoclonal anti-ZO-1/TJP1 antibody (1/500, #33–9100, Invitrogen, Carlsbad, USA). Secondary antibodies were donkey anti-mouse Alexafluor 488, 594 and donkey anti-rabbit Alexafluor 594, all from Invitrogen (1/1000). For nuclear staining, Hoechst 33258 (1/1000, Sigma-Aldrich, Taufkirchen, Germany) was used. For immunostaining of YAP, rabbit polyclonal YAP antibody (1/200, #4912, Cell Signaling, Danvers, USA) and DAB substrate (Roche Diagnostics Inc, Manheim, Germany) were used.

Samples were examined with a fluorescence microscope (BX51and DP80, Olympus, Tokyo, Japan) using cellSens software (Olympus, Tokyo, Japan). After H&E staining, the areas of living layer and stratum corneum were calculated using ImageJ 1.47 v (NIH, Bethesda, MD, USA).

### Electron-microscopic observation

Epidermal model samples for electron microscopy were minced (<0.5 mm^3^ pieces), fixed overnight in modified Karnovsky’s fixative, post-fixed in 2% aqueous osmium tetroxide or 0.2% ruthenium tetroxide, dehydrated in graded ethanol solutions, and embedded in Epon-epoxy mixture.

### Transepidermal water loss

Gravimetric transepidermal water loss (TEWL) was measured as described by Hanley *et al*.^21^, Inserts with epidermal models were placed dermis-side down onto silicon rubber plates and the lateral edges were sealed with petrolatum, so that water loss occurred only through the epidermal surface. Epidermis model sections were kept at ambient temperature (37 °C) and low humidity (<5%), and weighed for 2 hours. TEWL levels are reported as milligrams of water lost per square millimeter per hour. Epidermal model sections from four different subjects were used. We used 6 control (on flat membrane) models and 6 models on textile #255. Each sample was taken from an independent model.

### Statistics

The results are expressed as the mean ± SD. Statistical significance of differences between two groups was determined by a two-tailed Student’s t-test. In the case of more than 2 groups, statistical significance was determined by ANOVA with Tukey’s honestly significant difference (HSD), using KaleidaGraph (HULINKS, Tokyo, Japan). P-values less than 0.05 were considered significant.

## Electronic supplementary material

Supplement Computer simulation and Figures

## References

[1] Schäfer-Korting, M. & Schreiber, S. Use of skin equivalents for dermal absorption and toxicity. Roberts, M. S. and Walters, K. A. (eds) *Dermal Absorption and Toxicity Assessmen*t, Informa, USA pp141–159 (2008).

[2] Elias, P. M. Defensive functions of the stratum corneum: Integrative aspects. Elias, P. M. and Feingold, K. R. (eds) *Skin Barrier*, Taylor & Francis, USA, pp5–14 (2006).

[3] Sun R (2015). Lowered humidity produces human epidermal equivalents with enhanced barrier properties. Tissue Eng Part C Methods..

[4] Denda M (2014). Frontiers in epidermal barrier homeostasis - an approach to mathematical modeling of epidermal calcium dynamics. Exp Dermatol.

[5] Kobayashi Y, Sawabu Y, Kitahata H, Denda M, Nagayama M (2016). Mathematical model for calcium-assisted epidermal homeostasis. J Theor Biol.

[6] Kobayashi, Y. & Nagayama, M. Mathematical model of epidermal structure. R. S. Anderssen *et al*. (eds), *Applications* + *Practical Conceptualization* + *Mathematics* = *fruitful Innovation*, *Mathematics for Industry* 11, Springer Japan, pp121–126 (2016).

[7] Candi E, Schmidt R, Melino G (2005). The cornified envelope: a model of cell death in the skin. Nat Rev Mol Cell Biol..

[8] Brandner JM (2015). Epidermal tight junctions in health and disease. Tissue Barriers..

[9] Torkamani N, Rufaut NW, Jones L, Sinclair R (2016). Epidermal cells expressing putative cell markers in nonglabrous skin existing in direct proximity with the distal end of the arrector pili muscle. Stem Cells Int..

[10] Seirin LS (2016). Lateral inhibition-induced pattern formation controlled by the size and geometry of the cell. J Theor Biol..

[11] Hagiwara M (2016). An *in vitro*-in silico interface platform for spatiotemporal analysis of pattern formation in collective epithelial cells. Integr Biol (Camb)..

[12] Giangreco A, Goldie SJ, Failla V, Saintigny G, Watt FM (2010). Human skin aging is associated with reduced expression of the stem cell markers beta1 integrin and MCSP. J Invest Dermatol..

[13] Ghadially R, Brown BE, Sequeira-Martin SM, Feingold KR, Elias PM (1995). The aged epidermal permeability barrier. Structural, functional, and lipid biochemical abnormalities in humans and a senescent murine model. J Clin Invest..

[14] Halder G, Dupont S, Piccolo S (2012). Transduction of mechanical and cytoskeletal cues by YAP and TAZ. Nat Rev Mol Cell Biol..

[15] Porazinski S (2015). YAP is essential for tissue tension to ensure vertebrate 3D body shape. Nature..

[16] Dupont S (2011). Role of YAP/TAZ in mechanotransduction. Nature..

[17] Beverdam A (2013). Yap controls stem/progenitor cell proliferation in the mouse postnatal epidermis. J Invest Dermatol..

[18] Zhou K (2013). Actin-related protein2/3 complex regulates tight junctions and terminal differentiation to promote epidermal barrier formation. Proc Natl Acad Sci USA.

[19] Lee MJ, Ran Byun M, Furutani-Seiki M, Hong JH, Jung HS (2014). YAP and TAZ regulate skin wound healing. J Invest Dermatol..

[20] Randall MJ, Jüngel A, Rimann M, Wuertz-Kozak K (2018). Advances in the Biofabrication of 3D Skin *in vitro*: Healthy and Pathological Models. Front Bioeng Biotechnol..

[21] Hanley K, Rassner U, Elias PM, Williams ML, Feingold KR (1996). Epidermal barrier ontogenesis: maturation in serum-free media and acceleration by glucocorticoids and thyroid hormone but not selected growth factors. J Invest Dermatol..

