# Supplementary material for: Mathematical-model-guided development of full-thickness epidermal equivalent
Source: Sci Rep. 2018 Dec 20;8:17999. doi: 10.1038/s41598-018-36647-y (PMC6301960; doi:10.1038/s41598-018-36647-y)
Supplement: Supplementary file 1 — Supplement Computer simulation and Figures [file 41598_2018_36647_MOESM1_ESM.pdf]

Mathematical-model-guided development of full-thickness epidermal equivalent

Junichi Kumamoto[1], Shinobu Nakanishi[2], Mio Makita[2], Masaaki Uesaka[1], Yusuke Yasugahira[3], Yasuaki Kobayashi[4], Masaharu Nagayama[1], Sumiko Denda[2], *Mitsuhiro Denda[2]

1Research Institute for Electronic Science, Hokkaido University, Sapporo, Japan
2Shiseido Global Innovation Center, Yokohama, Japan
3Graduate School of Science, Hokkaido University, Sapporo, Japan
4Center for Simulation Sciences, Ochanomizu University, Tokyo, Japan

*Corresponding author: Mitsuhiro Denda, Shiseido Global Innovation Center, 2-2-1, Hayabuchi, Tsuzuki-ku, Yokohama, 224-8558, Japan. E-mail address: mitsuhiro.denda@to.shiseido.co.jp

Computer simulation. Our simulations are based on a previously proposed model[6], which was extended in the present work in order to incorporate the flattening of epidermal cells. The equations forming the complete model are given below. An attempt to incorporate the flattening of epidermal cells has been reported[21], but we employed a different approach.

In our model, a cell is represented as a spheroid. A cell i has ten variables ($\boldsymbol{x}_i(t)$, $r_i(t)$, $S_i(t)$, $\phi_i(t)$, $c_i(t)$, $P_i(t)$, $h_i(t)$, $v_i^{(p)}(t)$, $v_i^{(r)}(t)$, $\boldsymbol{n}_i(t)$): $\boldsymbol{x}_i(t)$ denotes the position of the cell, $r_i(t)$, the original radius of the cell, $S_i(t)$ the differentiation state variable, $\phi_i(t)$ the phase of the cell division cycle, $c_i(t)$ the intracytoplasmic calcium concentration, $P_i(t)$ the IP_3 concentration, $h_i(t)$ the inactivation factor, $v_i^{(p)}(t)$ and $v_i^{(r)}(t)$ the synthesized and secreted lipid quantity and $\boldsymbol{n}_i(t)$ the direction of the symmetry axis of the cell. The differentiation state of the cell is determined by $S_i(t)$: the cell is in the basal layer if $S_i(t) = 0$, a prickle cell if $0 < S_i(t) < S_g$, and a granular cell if $S_g \leq S_i(t) < S_c$. The cell cornifies when $S_c \leq S_i(t)$. Here, the threshold values S_g and S_c are set as $S_g = 3.0$ and $S_c = 22.0$. We fix the radius $r_i(t)$ to a constant value: $r_i = 2.0$. The length of the symmetry axis of the cell is r_i / α_i^2 and the other two axes are $\alpha_i r_i$. The parameter $\alpha_i = \alpha_i(S)$ is called the flattening rate of the cell, which depends on the differentiation state variable S_i and is governed by equation (1.1). We set $\alpha_{\mathrm{flat}}^{(\mathrm{max})} = 1.6$ and $\beta_{\mathrm{flat}} = 1.0$. The direction $\boldsymbol{n}_i(t)$ is always normalized and evolves according to equation (1.2), where we denote by $\boldsymbol{v}_i$ the velocity of the i-th cell. We set $\tau_{\boldsymbol{n}} = 0.001$ and $\alpha_{\boldsymbol{n}} = 1.0$.

The cell movement model. The position of the i-th cell $\boldsymbol{x}_i(t)$ is defined in a three-dimensional box $\Omega = [0, L_x] \times [0, L_y] \times [0, L_z]$, where we set $L_x = L_y = 60$ and $L_z = 100$. The movement of the cell depends on the cell type: the governing differential equations are equations (1.3), (1.4) and (1.5) when the cell is a stem cell, transit amplifying (TA) cell and supra-basal cell, respectively. Here we denote by Ω_j the set of all neighbouring cells and particles forming the basal membrane neighbouring the j-th cell. We consider the k-th cell as neighbouring the j-th cell if the distance between these cells is less than or equal to $1.2(\tilde{r}_j + \tilde{r}_k)$, where $\tilde{r}_j$ and $\tilde{r}_k$ are the effective radii of the j-th and k-th cells, respectively, defined as in (1.9). The set of all neighbouring basal membrane particles is denoted by Ω_j^{memb}.

$\boldsymbol{f}_{\mathrm{contact}}$ is the interacting force between neighbouring cells. This force is divided into a Lennard-Jones type repulsive force $\boldsymbol{f}_{\mathrm{LJ}}$ and an adhesion force $\boldsymbol{f}_{\mathrm{adhesion}}$, as shown in equation (1.6).

The Lennard-Jones type force $\boldsymbol{f}_{\mathrm{LJ}}$ is introduced in order to express the excluded volume effect and is given by equations (1.7) and (1.8). Here $\tilde{r}_j$ and $\tilde{r}_k$ are called the effective radii of cells j and k defined according to equation (1.9). This quantity is introduced because the cell is assumed to be spheroidal. We set $\varepsilon_{\mathrm{LJ}} = 0.02$ and $F_{\mathrm{max}} = 50$.

The adhesion force $\boldsymbol{f}_{\mathrm{adhesion}}$ is given by equations (1.10), (1.11) and (1.12). Here l^* is a parameter which gives the region encompassed by the adhesion force, and is chosen as $l^* = 1.2$. The coefficient K_{adhesion} depends on whether both cells are granular cells or not. The coefficients K_{total}

and $K_{\mathrm{desmosome}}$ are set as $K_{\mathrm{total}} = 7.0$ and $K_{\mathrm{desmosome}} = 0.21$, respectively.

The forces $\boldsymbol{f}^{(\mathrm{s})}_{\mathrm{basal}}$ and $\boldsymbol{f}^{(\mathrm{d})}_{\mathrm{basal}}$ represent the interaction force of the stem cell and the TA cell from the basal membrane, respectively. These forces are defined according to equations (1.13) and (1.14). We set $K_{\mathrm{s}} = 25.0$ and $K_{\mathrm{d}} = 5.0$.

The force $\boldsymbol{f}_{\mathrm{pair}}$ acts when a cell is undergoing division and represents the interaction force with the paired cell. The parameter χ_j is equal to 1 if cell division is underway and 0 otherwise. $\boldsymbol{f}_{\mathrm{pair}}$ is defined according to equation (1.15) and we set $K_{\mathrm{div}} = 5.0$, $R_{\mathrm{max}} = 2.0$ and $\varepsilon_{\mathrm{L}} = 0.14$.

The cell differentiation model. Cell differentiation is driven by the intracytoplasmic calcium concentration. The differentiation state variable S_i is governed by (1.16), which changes its behaviour according to whether the cell is cornified or not. c_{out} reflects the advance of differentiation in the presence of extra-cellular calcium and is set as $c_{\mathrm{out}} = 1.0 \times 10^{-3}$. We set $\beta_S = 1.5$, $\tau_s = 20$, $c_d = 0.1$ and $\tau_c = 20$. A cornified cell desquamates if S_i exceeds $S_{\mathrm{SC}} = 31.3$ and if the number of neighbouring cells is less than 17. It should be noted that we determine the correspondence between the time-scale in real skin and that in our simulation by assuming that a cornified cell desquamates 14 days after it has cornified.

The cell division model. Cell division is governed by the phase parameter $\phi_i(t)$, satisfying the differential equation (1.17). Cell division occurs with a certain probability if $\phi(t)$ exceeds the threshold S_{div}. We set the averaged cell cycle as 4 days in TA cells and 18 days in stem cells.

When the i-th cell is divided into two cells labelled j and k at time t_0, the initial positions are the same: $\boldsymbol{x}_j(t_0) = \boldsymbol{x}_k(t_0)$. We assume that the division is complete if $\| \boldsymbol{x}_j(t) - \boldsymbol{x}_k(t) \| > 1.8R_{\mathrm{max}}$.

A stem cell can reproduce an infinite number of times, while a TA cell can reproduce only a finite number of times. We set this maximum reproducing number of TA cells to be 10.

We also assume that a stem cell is tightly bound to the basal membrane. On the other hand, a TA cell is comparatively loosely bound and can migrate to the supra-basal layer. We consider that once a TA cell leaves the basal layer, it starts its differentiation process.

The lipid dynamics model. The synthesized and secreted lipid quantities $v_i^{(\mathrm{p})}$ and $v_i^{(\mathrm{r})}$ satisfy equations (1.19) and (1.20). The parameters are chosen as follows: $v_{max}^{(p)} = 1$, $c_0 = 0.45$, $k^{(\mathrm{p})}=0.6$, $\sigma_{r1} = 0.1$, $\sigma_{r2} = 0.1$ and $k^{(\mathrm{r})} = 0.2$, $\tau_{\mathrm{p}} = 1.0$, $\tau_{\mathrm{s}} = 1.0$.

The extracellular stimulant dynamics model. In (6), we assumed that an extra-cellular stimulant is released by a cell when it cornifies and that this stimulant induces calcium excitation. The concentration of this stimulant (,) is governed by the diffusion equation (1.21). The parameters are chosen as follows: $d_b = 0.00126$, $K_{bb} = 0.025$, $S_1 = 21.5$ and $S_2 = 24$. We impose

the periodic boundary condition in the x and y directions and the Neumann condition in the z direction to solve this partial differential equation.

The calcium dynamics model. The dynamics of $c_i(t)$, $P_i(t)$ and $h_i(t)$ is governed by the equations (1.21). Here $A(t, x)$ denotes the extracellular ATP concentration, and w_{ij} the activity of the gap-junctions between cell i and cell j. The constants in this model are chosen as follows: $d_A = 1.4$, $K_{aa} = 0.5$, $K_{ac} = 0.002$, $d_c = 0.01$, $\delta_I = 1.5$, $K_f = 8.1$, $\mu_0 = 0.567$, $\mu_1 = 0.1$, $K\mu = 0.05$, $\alpha_0 = 0.11$, $K_1 = 0.7$, $\gamma = 2.0$, $K_\gamma = 0.1$, $\beta = 0.02$, $K_{bc} = 0.48$, $H_b = 0.01$, $H_0 = 0.5$, $K_2 = 0.7$, $\varepsilon_{w0} = 0.1$, $w_{d0} = 0.1$, $\tau_g = 0.2$, $\tau_s = 1.0$, $\delta_t = 1.0$, $\delta_I = 1.5$, $k_g = 4.0$, $k_s = 6.0$ and $\delta_k = 1.0$. When calculating $A(t, x)$, we impose the periodic boundary condition in the x and y directions and the Neumann boundary condition in the z direction.

Basement membrane. The basement membrane is expressed as a set of particles whose radii are all equal to 1.0. In the flat case, the membrane is fixed at the plane $z = 0$; in the curved case, the coordinate (x, y, z) of the membrane particle satisfies $z = 5.0 \cos(6\pi x/L_x) \cos(6\pi y/L_y)$. The number of membrane particles is fixed to 16560.

Numerical simulation. For numerical simulation, we set the time step $\Delta t = 0.01$. For particle dynamics, we impose the periodic boundary condition in the x and y directions. We confirmed that L_z is sufficiently large that the uppermost particles desquamate before reaching $z = L_z$. The timescale of calcium dynamics is far faster than that of cell division, migration and differentiation. Accordingly, we calculate the dynamics of cells and the stimulant $B(t, x)$ with the calcium levels kept constant. The calcium dynamics is calculated when a certain number of cells (we choose 20 in our simulations) cornify. In calcium calculation, we regarded $\boldsymbol{x}_i(t)$, $S_i(t)$, $\phi_i(t)$, $B(t, \boldsymbol{x})$ as constant and the calculation is continued until the system reaches a steady state. The two phases of cell dynamics calculation and calcium calculation are repeated.

1 Full model equations

Here and henceforth, $\chi(r)$ denotes the Heaviside step function:

$$\chi(r) := \begin{cases} 1, & r > 0, \\ 0, & r \leq 0. \end{cases}$$

and $x_+ = \max(x, 0)$.

1.1 Cell deformation model

The flattening rate is described as follows:

$$\alpha_i = \alpha(S_i) := \frac{1 + \alpha_{\text{flat}}^{(\max)} \beta_{\text{flat}} S_i^2}{1 + \beta_{\text{flat}} S_i^2}. \tag{1.1}$$

The deformation direction $\boldsymbol{n}_i$ of the i-th cell is given by the following equation:

$$\tau_{\boldsymbol{n}} \frac{d}{dt} \boldsymbol{n}_i = \begin{cases} (\boldsymbol{v}_i(t) - \boldsymbol{n}_i(t)), & \text{if } \|\boldsymbol{v}_i(t) - \boldsymbol{n}_i(t)\| < \alpha_{\boldsymbol{n}}, \\ \alpha_{\boldsymbol{n}} \dfrac{\boldsymbol{v}_i(t) - \boldsymbol{n}_i(t)}{\|\boldsymbol{v}_i(t) - \boldsymbol{n}_i(t)\|}, & \text{if } \|\boldsymbol{v}_i(t) - \boldsymbol{n}_i(t)\| \geq \alpha_{\boldsymbol{n}}, \end{cases} \tag{1.2}$$

where $\boldsymbol{v}_i(t)$ is the velocity of the i-th cell, which is given by the cell movement model. Then a cell C_i is described as a spheroid:

$$\begin{aligned} C_i = \Bigg\{ & (x, y, z)\,; \\ & \frac{1}{\alpha_i^2 r_i^2} \left((x - x_i) \sin\theta_i - (y - y_i) \cos\theta_i \right)^2 \\ & + \frac{1}{\alpha_i^2 r_i^2} \left((x - x_i) \cos\theta_i \sin\varphi_i + (y - y_i) \sin\theta_i \sin\varphi_i - (z - z_i) \cos\varphi_i \right)^2 \\ & + \frac{\alpha_i^4}{r_i^2} \left((x - x_i) \cos\theta_i \cos\varphi_i + (y - y_i) \sin\theta_i \cos\varphi_i + (z - z_i) \sin\varphi_i \right)^2 \leq 1 \Bigg\}, \end{aligned}$$

where $\boldsymbol{x}_i = \begin{pmatrix} x_i \\ y_i \\ z_i \end{pmatrix}$ and $\boldsymbol{n}_i = \begin{pmatrix} \cos\theta_i \cos\varphi_i \\ \sin\theta_i \cos\varphi_i \\ \sin\varphi_i \end{pmatrix}$.

1.2 Cell movement model

$$\frac{d\boldsymbol{x}_j^{(\text{s})}}{dt} = \sum_{k \in \Omega_j} \boldsymbol{f}_{\text{contact}}(\boldsymbol{x}_j^{(\text{s})}, \boldsymbol{x}_k) + \sum_{k \in \Omega_j^{\text{memb}}} \boldsymbol{f}_{\text{basal}}^{(\text{s})}(\boldsymbol{x}_j^{(\text{s})}, \boldsymbol{x}_k^{(\text{memb})}) + \chi_j \boldsymbol{f}_{\text{pair}}(\boldsymbol{x}_j^{(\text{s})}, \boldsymbol{x}_{j'}), \tag{1.3}$$

$$\frac{d\boldsymbol{x}_j^{(\text{d})}}{dt} = \sum_{k \in \Omega_j} \boldsymbol{f}_{\text{contact}}(\boldsymbol{x}_j^{(\text{d})}, \boldsymbol{x}_k) + \sum_{k \in \Omega_j^{\text{memb}}} \boldsymbol{f}_{\text{basal}}^{(\text{d})}(\boldsymbol{x}_j^{(\text{d})}, \boldsymbol{x}_k^{(\text{memb})}) + \chi_j \boldsymbol{f}_{\text{pair}}(\boldsymbol{x}_j^{(\text{d})}, \boldsymbol{x}_{j'}), \tag{1.4}$$

$$\frac{d\boldsymbol{x}_j^{(\text{supra})}}{dt} = \sum_{k\in\Omega_j} \boldsymbol{f}_{\text{contact}}(\boldsymbol{x}_j^{(\text{supra})}, \boldsymbol{x}_k), \tag{1.5}$$

where

$$\begin{aligned}\boldsymbol{f}_{\text{contact}}(\boldsymbol{x}_j, \boldsymbol{x}_k) = \boldsymbol{f}_{\text{LJ}}(\boldsymbol{x}_j, \boldsymbol{x}_k)\chi\left(1 - \frac{\|\boldsymbol{x}_j - \boldsymbol{x}_k\|}{r_j + r_k}\right) \\ + \boldsymbol{f}_{\text{adhesion}}(\boldsymbol{x}_j, \boldsymbol{x}_k, K_{\text{adhesion}})\chi\left(\frac{\|\boldsymbol{x}_j - \boldsymbol{x}_k\|}{r_j + r_k} - 1\right).\end{aligned} \tag{1.6}$$

$$\boldsymbol{f}_{\text{LJ}}(\boldsymbol{x}_j, \boldsymbol{x}_k) := \begin{cases} \boldsymbol{f}_{\text{LJ}}^{(\text{old})}(\boldsymbol{x}_j, \boldsymbol{x}_k), & \text{if } \left|\boldsymbol{f}_{\text{LJ}}^{(\text{old})}(\boldsymbol{x}_j, \boldsymbol{x}_k)\right| \le F_{\max}, \\ F_{\max}\dfrac{\boldsymbol{x}_j - \boldsymbol{x}_k}{\|\boldsymbol{x}_j - \boldsymbol{x}_k\|}, & \text{otherwise.} \end{cases} \tag{1.7}$$

$$\boldsymbol{f}_{\text{LJ}}^{(\text{old})}(\boldsymbol{x}_j, \boldsymbol{x}_k) := \frac{4\epsilon_{\text{LJ}}}{\|\boldsymbol{x}_j - \boldsymbol{x}_k\|}\left[\left(\frac{\widetilde{r}_j + \widetilde{r}_k}{\|\boldsymbol{x}_j - \boldsymbol{x}_k\|}\right)^{12} - \left(\frac{\widetilde{r}_j + \widetilde{r}_k}{\|\boldsymbol{x}_j - \boldsymbol{x}_k\|}\right)^{6}\right]\frac{\boldsymbol{x}_j - \boldsymbol{x}_k}{\|\boldsymbol{x}_j - \boldsymbol{x}_k\|}. \tag{1.8}$$

$$\begin{aligned}\widetilde{r}_j &:= \sqrt{\frac{r_j^2}{\alpha_j^4}\cos^2\theta_j + \alpha_j^2 r_j^4 \sin^2\theta_j}, \\ \theta_j &:= \arccos\left(\frac{(\boldsymbol{x}_k - \boldsymbol{x}_j)}{\|\boldsymbol{x}_k - \boldsymbol{x}_j\|}\cdot \boldsymbol{n}_j\right).\end{aligned} \tag{1.9}$$

$$\begin{aligned}&\boldsymbol{f}_{\text{adhesion}}(\boldsymbol{x}_j, \boldsymbol{x}_k, K_{\text{adhesion}}) \\ &= \begin{cases} -K_{\text{adhesion}} f\left(\dfrac{\|\boldsymbol{x}_j - \boldsymbol{x}_k\|}{\widetilde{r}_j + \widetilde{r}_k} - 1\right)\dfrac{\boldsymbol{x}_j - \boldsymbol{x}_k}{\|\boldsymbol{x}_j - \boldsymbol{x}_k\|}, & \text{if } \dfrac{\|\boldsymbol{x}_j - \boldsymbol{x}_k\|}{\widetilde{r}_j + \widetilde{r}_k} < l^*, \\ 0, & \text{otherwise,} \end{cases}\end{aligned} \tag{1.10}$$

where

$$f(r) = r - \frac{1}{(l^* - 1)^2} r^3, \tag{1.11}$$

$$K_{\text{adhesion}} = \begin{cases} K_{\text{total}}, & \text{if } S_j \ge S_g \text{ and } S_k \ge S_g, \\ K_{\text{desmosome}}, & \text{otherwise,} \end{cases} \tag{1.12}$$

$$\begin{aligned}\boldsymbol{f}_{\text{basal}}^{(\text{s})}(\boldsymbol{x}_j, \boldsymbol{x}_k) = \boldsymbol{f}_{\text{LJ}}(\boldsymbol{x}_j, \boldsymbol{x}_k)\chi\left(1 - \frac{\|\boldsymbol{x}_j - \boldsymbol{x}_k\|}{r_j + r_k}\right) \\ - K_s\frac{\boldsymbol{x}_j - \boldsymbol{x}_k}{\|\boldsymbol{x}_j - \boldsymbol{x}_k\|}\left(\frac{\|\boldsymbol{x}_j - \boldsymbol{x}_k\|}{r_j + r_k} - 1\right)\chi\left(\frac{\|\boldsymbol{x}_j - \boldsymbol{x}_k\|}{r_j + r_k} - 1\right),\end{aligned} \tag{1.13}$$

$$\begin{aligned}\boldsymbol{f}_{\text{basal}}^{(\text{d})}(\boldsymbol{x}_j, \boldsymbol{x}_k) = \boldsymbol{f}_{\text{LJ}}(\boldsymbol{x}_j, \boldsymbol{x}_k)\chi\left(1 - \frac{\|\boldsymbol{x}_j - \boldsymbol{x}_k\|}{r_j + r_k}\right) \\ + \boldsymbol{f}_{\text{adhesion}}(\boldsymbol{x}_j, \boldsymbol{x}_k, K_d)\chi\left(\frac{\|\boldsymbol{x}_j - \boldsymbol{x}_k\|}{r_j + r_k} - 1\right),\end{aligned} \tag{1.14}$$

$$\begin{aligned}\boldsymbol{f}_{\text{pair}}(\boldsymbol{x}_j, \boldsymbol{x}_{j'}) &= -K_{\text{div}}\frac{\boldsymbol{x}_j - \boldsymbol{x}_{j'}}{\|\boldsymbol{x}_j - \boldsymbol{x}_{j'}\|}\left(\|\boldsymbol{x}_j - \boldsymbol{x}_{j'}\| - \ell(t)\right), \\ \frac{d}{dt}\ell(t) &= \epsilon_{\text{L}}(2R_{\max} - \ell(t)).\end{aligned} \tag{1.15}$$

1.3 Cell differentiation model

$$
\begin{aligned}
\tau_S \frac{d}{dt} S_i &= c_{\text{out}} + \beta_S (c_i - c_0)_+, \quad && 0 \le S_i < S_c, \\
\tau_S \frac{d}{dt} S_i &= c_d, && S_i \ge S_c.
\end{aligned}
\tag{1.16}
$$

1.4 Cell division model

$$
\tau_\phi \frac{d}{dt} \phi_i = \phi_0 + \beta_\phi (c_i - c_0)_+. \tag{1.17}
$$

1.5 Lipid dynamics model

$$
\begin{cases}
\tau_p \dfrac{d}{dt} v_i^{(\mathrm{p})} & = (v_{\max}^{(\mathrm{p})} - v_i^{(p)}) f^{(p)}(c_i, S_i) - v_i^{(p)} f^{(r)}(c_i, S_i), \\
\tau_r \dfrac{d}{dt} v_i^{(r)} & = v_i^{(p)} f^{(r)}(c_i, S_i),
\end{cases}
\tag{1.18}
$$

where

$$
f^{(p)}(c_i, S_i) = \frac{k^{(\mathrm{p})}}{4} \left(1 + \tanh\left(\frac{S_i - S_g}{\sigma_{r1}}\right)\right) \left(1 + \tanh\left(\frac{c_0 - c_i}{\sigma_{r2}}\right)\right), \tag{1.19}
$$

$$
f^{(r)}(c_i, S_i) = \frac{k^{(\mathrm{r})}}{4} \left(1 + \tanh\left(\frac{S_i - S_g}{\sigma_{r1}}\right)\right) \left(1 + \tanh\left(\frac{c_i - c_0}{\sigma_{r2}}\right)\right). \tag{1.20}
$$

1.6 Extra-cellular stimulant dynamics model

$$
\frac{\partial B}{\partial t} = d_b \Delta B - K_{bb} B + \sum_{i=1}^{N} F_B(S_i, \boldsymbol{x}_i), \tag{1.21}
$$

where

$$
F_B(S_i, \boldsymbol{x}_i) = \chi(S_i - S_1) \chi(S_2 - S_i) \chi(r - \|\boldsymbol{x} - \boldsymbol{x}_i\|).
$$

1.7 Calcium dynamics model

$$
\begin{cases}
\dfrac{\partial A}{\partial t} & = d_A \Delta A - K_{aa} A + \displaystyle\sum_{i=1}^{N} G\left(\boldsymbol{x}, \boldsymbol{x}_i, \frac{dc_i}{dt}\right), \\
\dfrac{dP_i}{dt} & = \displaystyle\sum_{j \in \Lambda_i} d_P w_{ij} I_n(S_i)(P_j - P_i) + F_P(A, S_i) - K_{pp} P_i, \\
\dfrac{dc_i}{dt} & = \displaystyle\sum_{j \in \Lambda_i} d_c w_{ij} I_n(S_i)(c_j - c_i) + F_c(P_i, c_i, h_i, B), \\
\dfrac{dh_i}{dt} & = \dfrac{1}{\tau_h(S_{k_i})} F_h(c_i, h_i), \\
\dfrac{dw_{ij}}{dt} & = F_w(w_{ij}, c_i, c_j),
\end{cases}
\tag{1.22}
$$

where

$$\boldsymbol{x},\ \boldsymbol{x}_i \in \Omega = [0, L_x] \times [0, L_y] \times [0, L_z],\ t > 0,\ i \in \{1, 2, \ldots N(t)\}.$$

$$G\left(\boldsymbol{x}, \boldsymbol{x}_i, \frac{dc_i}{dt}\right) = K_{ac}\chi(r - \|\boldsymbol{x} - \boldsymbol{x}_i\|)\chi\left(\frac{dc_i}{dt}\right), \tag{1.23}$$

$$F_c(P, c, h, B) = K_f\left(\mu_0 + \frac{\mu_1 P}{K_\mu + P}\right)\left(\alpha_0 + \frac{(1-\alpha_0)c}{K_1 + c}\right)h - \frac{\gamma c}{K_\gamma + c} + \beta + \frac{K_{bc}B^2}{H_b + B^2}, \tag{1.24}$$

$$F_P(A, S_i) = K_{pa}(S_i)\frac{A}{H_0 + A}, \qquad F_h(c, h) = \frac{K_2^2}{K_2^2 + c^2} - h, \tag{1.25}$$

$$F_w(w_{ij}, c_i, c_j) = -w_{ij} + \frac{1}{2}\left(1 + \tanh\left(\frac{w_{d0} - |c_i - c_j|}{\varepsilon_{w0}}\right)\right), \tag{1.26}$$

$$\tau_h(S_i) = \tau_g + \frac{\tau_s - \tau_g}{2}\left(1 + \tanh\left(\frac{S_g - S_i}{\delta_\tau}\right)\right), \tag{1.27}$$

$$I_n(S_i) = \frac{1}{2}\left(1 + \tanh\left(\frac{S_i - S_g}{\delta_I}\right)\right), \tag{1.28}$$

$$K_{pa}(S_i) = k_g + \frac{k_s - k_g}{2}\left(1 + \tanh\left(\frac{S_g - S_i}{\delta_k}\right)\right). \tag{1.29}$$

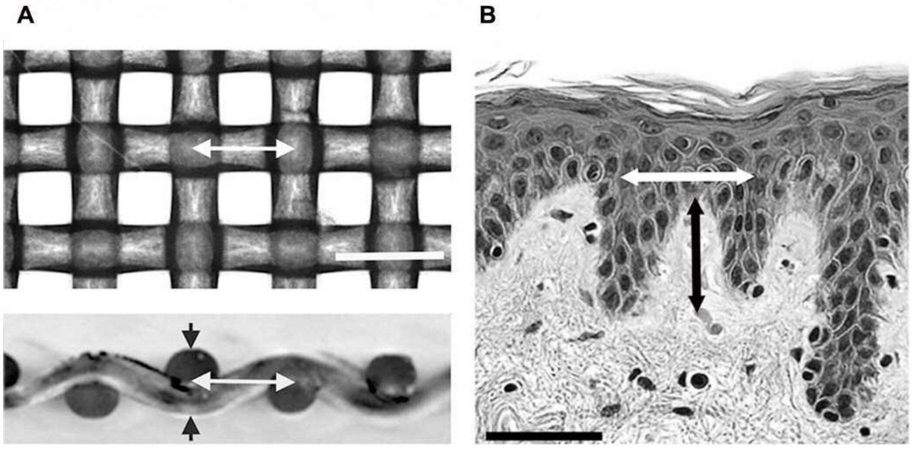

Supplement Figure 1. Evaluation of thickness and inter-fiber interval (undulation) of textile samples and human skin papillary layer. (**A**) Overhead view of textile #255 (upper image) and cross section of the textile (lower image). White arrows indicate the inter-fiber interval (undulation) of the textile and the thickness of the undulation is indicated by black arrowheads. White bar = 100 μm. (**B**) Corresponding parameters of human epidermis. The undulation interval is indicated by a white arrow and the thickness is indicated by a black arrow.

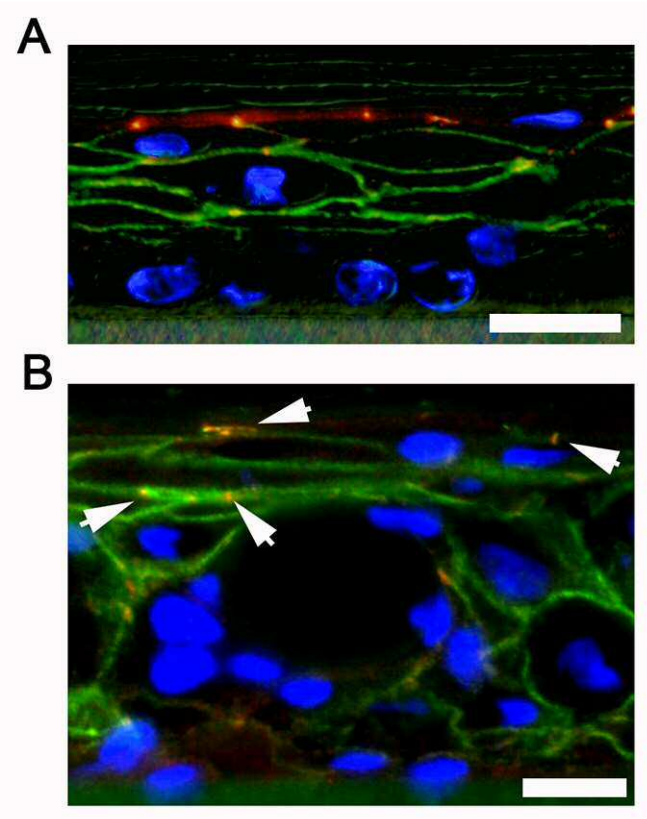

Supplement Figure 2 Merged images of immunostaining with tight junction markers ZO-1 (Red) and claudin 1 (Green) of control (A) and #300 textile (B) models. Bars = 50 μm. In the control, ZO-1 was expressed in the uppermost layer and claudin 1 was expressed in the cell membranes throughout the layers (A). In the case of #300, claudin 1 was expressed in the cell membranes throughout the layers and a little ZO-1 was expressed in the upper layer of the epidermis (B: arrows).

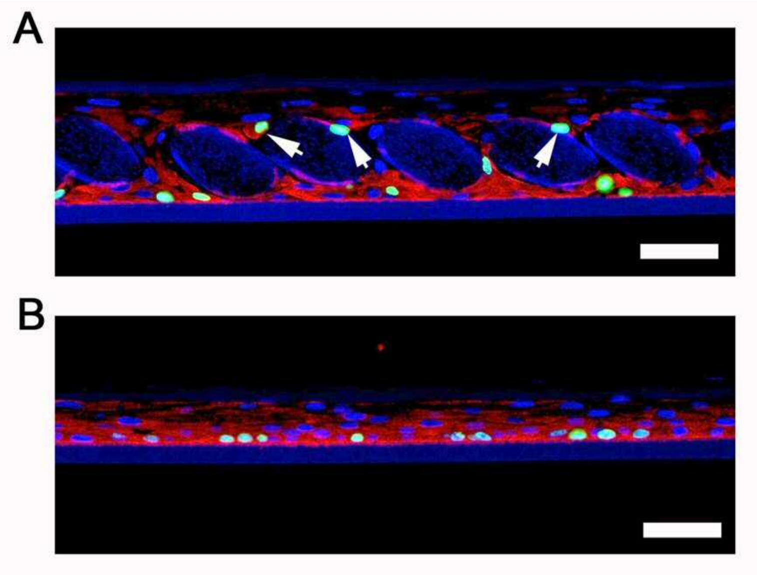

Supplement figure 3. Merged images of co-immunostaining with K14 (red, basal cell marker, anti-cytokeratin 14 antibody, 1/1000, clone RCK107, # MAB3232, EMD Millipore, Burlington, USA) and BrdU (green) of #255 textile (A) and control (B) models. BrdU-positive cells were localized at the K14-positive area, even on top of the #255 fibers (A: arrows). Bars = 50 µm.

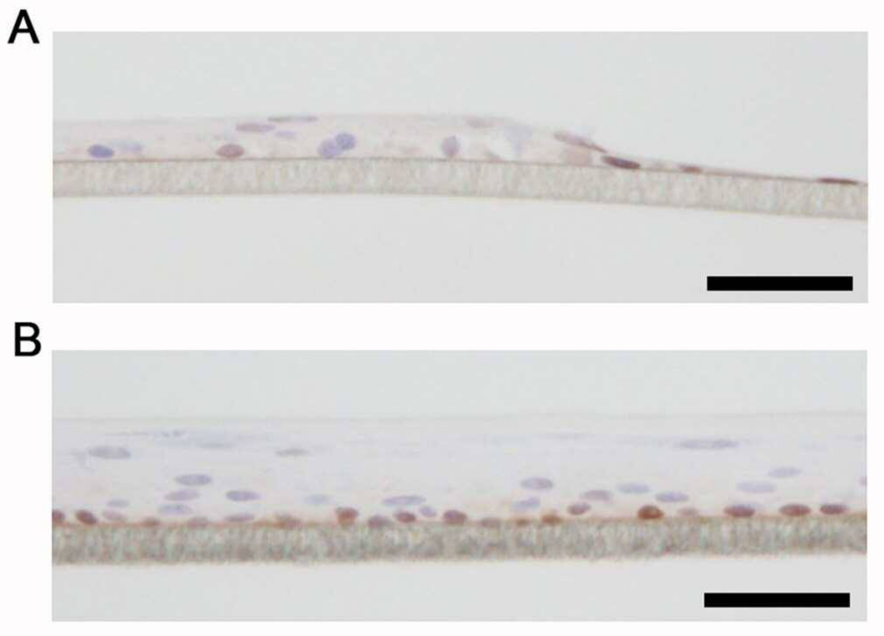

Supplement figure 4. Effect of YAP siRNA on the control epidermal model. Control epidermal models after YAP siRNA (A) and control siRNA (B) treatment were stained with YAP antibody (brown color). RT-PCR showed that YAP siRNA decreased YAP expression by 87±2%. The YAP siRNA-treated model showed few YAP-stained nuclei, and structure formation was disrupted (A). The control siRNA-treated model exhibited YAP-positive nuclei at the basal layer (B). Bars = 50 μm.
